# Association of tympanostomy tubes with future assistive hearing devices–a population based study

**DOI:** 10.1186/s12887-020-1977-6

**Published:** 2020-02-18

**Authors:** Jason A. Beyea, Bonnie Cooke, Emily Rosen, Paul Nguyen

**Affiliations:** 1 Department of Otolaryngology, Kingston Health Sciences Centre, Queen’s University, Kingston, 144 Brock Street, Kingston, Ontario K7L 5G2 Canada; 2 ICES Adjunct Scientist ICES Queen’s, Queen’s University School of Medicine, Kingston, 144 Brock Street, Kingston, Ontario K7L 5G2 Canada; 3 Department of Speech Language Pathology and Audiology, Hotel Dieu Hospital, Kingston, ON Canada

**Keywords:** Hearing loss, Tympanostomy tubes, Hearing aids, Otitis media, Ear surgery, Outcomes, Implantable hearing devices

## Abstract

**Background:**

Ear (tympanostomy) tube (TT) placement is a common ambulatory surgery in children. Despite the commonality of this treatment, the long-term effects are unknown. The objective of this study was to determine the rate of permanent hearing loss, as measured by use of a rehabilitative hearing device.

**Methods:**

A retrospective comprehensive population-based cohort study was performed, evaluating all hospitals in the Canadian province of Ontario. Three cohorts of children were constructed: TT – at least one ear tube procedure (*n* = 193,880), No-TT –recurrent visits to a physician for middle ear disease, did not undergo ear tubes (*n* = 203,283), and Control – an age/sex matched group who had not undergone ear tubes and who didn’t have repeat physician visits for middle ear disease (*n* = 961,168). The main outcome measures were risk and odds ratio (OR) of rehabilitative hearing devices.

**Results:**

The TT cohort had a higher risk of obtaining a hearing aid (OR 4.53 vs. No-TT, *p* < 0.001; OR 10.81 vs. Control, *p* < 0.001), an FM system (OR 3.84 vs. No-TT, *p* < 0.001; OR 15.13 vs. Control, *p* < 0.001), and an implanted bone conduction device (OR 5.08 vs. No-TT, *p* < 0.001; OR 15.67 vs. Control, *p* < 0.001).

**Conclusions:**

An association between ear tube placement and long-term need for a rehabilitative hearing device was found. This association warrants future prospective research in this area.

## Background

Ear (tympanostomy) tube (TT) placement is a common ambulatory surgery that children undergo [1]. Persistent otitis media with effusion (OME) and recurrent acute otitis media (AOM) are the most common indications for which TT placement is performed [2].

Insertion of TT has been shown to be associated with development of eardrum pathologic abnormalities, including segmental atrophy, perforation, and cholesteatoma [3]. Furthermore, studies exist supporting higher hearing thresholds in children managed with TT insertion versus those managed conservatively/medically [4, 5]. However, others have not found evidence of hearing loss post-TT insertion [6, 7].

The consistent feature of these studies is the small sample size, and as such, the risk of type-2 error is present. The current study was designed to evaluate in a large comprehensive population long-term hearing loss in TT patients of a severity that merited a rehabilitative hearing device, and to compare this population to patients with recurrent middle ear disease managed without TT, and to healthy age/sex-matched controls.

## Methods

### Ethical considerations

Queen’s University Health Sciences & Affiliated Teaching Hospitals Research Ethics Board approved this study, project #6017300.

### Participants

Ontario has a population of over 14 million residents, the largest Canadian province. Canadian hospitals must report all same-day and inpatient operative procedures. The Canada Health Act governs the Canadian healthcare system, which is a publically funded and administered. This Act mandates comprehensive universal coverage for all medically necessary services, including TT and bone-conducting hearing aids. Private health insurance for these procedures is prohibited. Furthermore, all residents are eligible (based on medical necessity) for partial financial coverage of assistive hearing devices (such as hearing aids and FM systems) through the Assistive Devices Program (ADP). These surgical procedures and assistive devices are available to all persons equally. The present study defined children as persons aged 0 to 18 years. Exclusion criteria were: a valid patient identifier was not available in the dataset, if age/sex information was not available, or if they had undergone a TT procedure (Ontario Health Insurance Plan feecode Z914) in the 2 years prior to January 1, 1994. To further characterize the cohorts, the diagnosis of cleft palate/lip was sought (Table 1), using a window of 2 years prior to the index date and 5 years following the index date.

**Table 1:** Ontario Health Insurance Plan (OHIP) Diagnosis and Billing Administrative Codes

	Diagnosis Code	Billing Code
Eustachian Tube Dysfunction/Serous Otitis Media	381	
Eustachian Tube Dysfunction/Suppurative Otitis Media	382	
Cleft Palate/Lip	789	
Myringotomy with insertion of ventilating (tympanostomy) tube		Z914
Implantable bone conduction hearing aid insertion		E346

### Data sources and data linkage

The data for this study was obtained from administrative datasets housed at ICES. ICES is an independent, non-profit research organization funded by the Ontario Ministry of Health and Long Term Care whose comprehensive data holdings include all health care related events for the population of Ontario. The Registered Persons Database (RPDB) contains demographic information for all Ontario residents who are eligible for the Ontario Health Insurance Plan (OHIP). RPDB data is maintained by the Ontario Ministry of Health and Long Term Care. RPDB data includes: health card number, date of birth, gender, address, and deceased date (if applicable). At ICES, all personal identifying information in RPDB is removed. An anonymous unique identifier, the ICES Key Number (IKN) is generated from each health number. The IKN is used to link data sources within ICES. Diagnosis and fee-for-service claims submitted by physicians, and paid by the universal health care system, are contained within the OHIP database. The Assistive Devices Program (ADP) database contains (among many other variables) information regarding attainment of an assistive hearing device, including a hearing aid and/or FM system.

### Intervention

Three cohorts were constructed and evaluated at ICES. The **TT cohort** was constructed of all children who had received at least one TT surgery during the study period (January 1, 1994 to October 31, 2013). The **No-TT cohort** had more than five visits to a physician with an Eustachian tube dysfunction diagnosis (Serous Otitis Media or Suppurative Otitis Media) within 1 year (Table 1). These No-TT cohort patients did not undergo TT surgery, and all children who met this criterion were included in this cohort. The purpose of this criterion was to capture children with considerable ongoing middle ear dysfunction, while avoiding capture of children with occasional middle ear disease. The Eustachian tube dysfunction diagnostic codes (Table 1) were validated in the TT cohort – based on an average of five visits in the year prior to TT placement. There is also construct validity, since based on current indications for TT surgery [8], many children with five or more visits per year to primary care for AOM or middle ear disease would be considered for TT surgery. A **Control cohort** was created, by matching to the TT cohort 5-to-1 by birth year and sex, Ontario children who had two or less uses of these Eustachian tube dysfunction diagnostic codes in any 1 year period. In clinical practice, these are children who it would be very unlikely that TT surgery would be presented as a management option. In the year prior to the TT surgery in the TT cohort, less than 30% of patients had these codes used 2 or less times. Patients were also excluded from the No-TT and Control cohorts if they had undergone a TT surgery between January 1992 and March 2016. The creation of these three cohorts permitted determination of risks of assistive hearing devices in patients who underwent TT surgery, patients with recurrent middle ear disease managed non-surgically, and controls.

### Outcomes

In the three cohorts, OHIP diagnostic and billing codes for ear disease and ear surgical procedures performed (Table 1), and obtainment of an assistive hearing device from the ADP claims database, were collected and evaluated.

### Statistical analysis

Descriptive statistics of patient characteristics were obtained. Logistic regression analyses were then utilized to compare ear surgical procedures performed (i.e., bone-conducting hearing aids) and obtainment of an assistive hearing device (i.e., unilateral/bilateral hearing aids and FM system) claimed in patients between the TT cohort and the No-TT cohort, and between the TT cohort and the Control cohort.

Statistical analyses were performed at ICES using SAS Enterprise Guide software, version 7.1 (SAS Institute, Cary, NC). Two-sided statistical significance was a *p*-value< 0.05.

## Results

Three cohorts of children were studied: TT cohort (193,880 children), No-TT cohort (203,283 children), and Control cohort (961,168 children) (Table 2). Both the TT and No-TT cohort had an average above 5 episodes of physician-diagnosed Eustachian tube dysfunction in the year preceding the index date.

**Table 2:** Cohort Characteristics

	TT Cohort	No-TT Cohort	Control Cohort
Sample Size	193,880	203,283	961,168
Age (years) at Index Date^a^ (Mean ± SD)	3.61 ± 2.85	2.43 ± 2.65^*^	3.08 ± 2.88^*^
Sex (% female)	39.29	45.21^*^	39.30
Episodes of Physician-diagnosed Eustachian Tube Dysfunction (Mean ± SD, per year)	5.11 ± 3.91^b^	5.06 ± 0.37^b*^	0.14 ± 0.40^b*^

^a^Index dates are the date of first TT procedure for the TT cohort, latest diagnosis date of > 5 episodes in 1-year period for the No-TT cohort, and date of first TT procedure of the matched children from TT cohort for the Control cohort

^b^in 1-year prior to index date

^*^p < 0.001 versus TT cohort

*TT* Tympanostomy Tube

*SD* Standard Deviation

Occurrence and odds ratios of assistive hearing devices were compared in the three cohorts (Table 3). While overall occurrences were low, the large size of the cohorts permitted meaningful comparisons. Odds ratios comparing the TT cohort with the Control cohort were high for all assistive hearing devices. In addition, the odds ratios of the TT cohort versus the No-TT cohort were consistently around 4 for all assistive hearing devices.

**Table 3:** Occurrence and Odds Ratio (OR) [with 95% Confidence Interval (CI)] of Assistive Hearing Devices

	TT Cohort (%)	No-TT Cohort (%)	Control Cohort (%)	TT vs. No-TT [OR (95% CI)]	TT vs. Control [OR (95% CI)]
Implanted bone conduction device	0.032	0.006	0.002	5.08 (2.80–9.23)^*^	15.67 (9.47–25.91)^*^
Unilateral hearing aid	0.341	0.081	0.025	4.21 (3.55–5.00)^*^	13.74 (11.85–15.94)^*^
Bilateral hearing aid^a^	0.647	0.139	0.067	4.69 (4.12–5.34)^*^	9.70 (8.82–10.67)^*^
Unilateral or bilateral hearing aid	0.988	0.220	0.092	4.53 (4.09–5.02)^*^	10.81 (9.98–11.71)^*^
FM system hearing device	0.121	0.031	0.008	3.84 (2.91–5.06)^*^	15.13 (11.69–19.57)^*^
Any hearing rehabilitation device	1.035	0.236	0.097	4.43 (4.01–4.89)^*^	10.76 (9.95–11.63)^*^

^a^Patients who received bilateral hearing aids are not counted in the unilateral hearing aid data

^*^p < 0.001

**Fig. 1:**
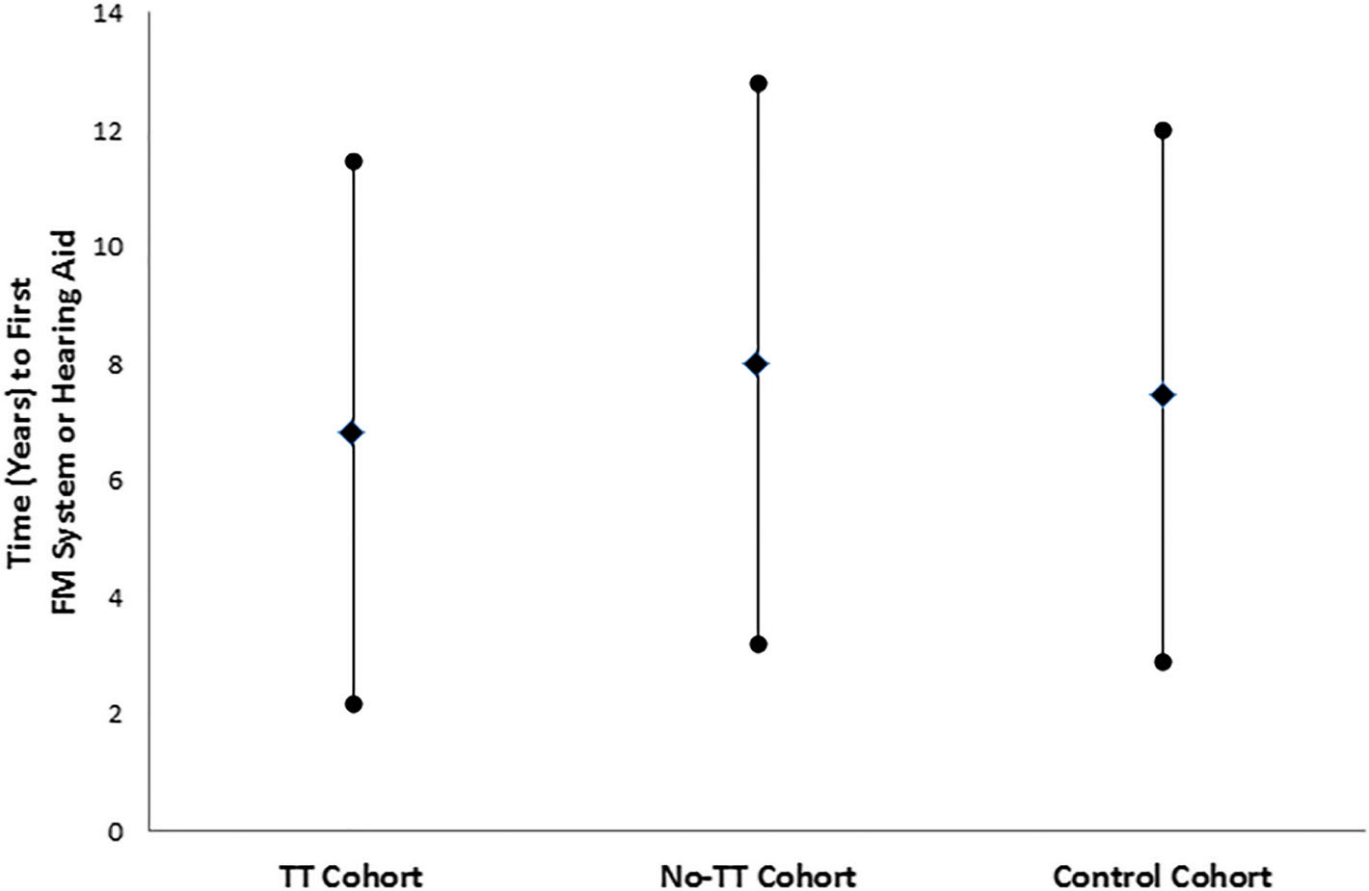
Time (years) (Mean ± SD) from index date to first FM system or hearing aid in the three cohorts. Time was created for cohort patients whose index date was after April 1, 1999 due to data limitations in the ADP database

**Fig. 2:**
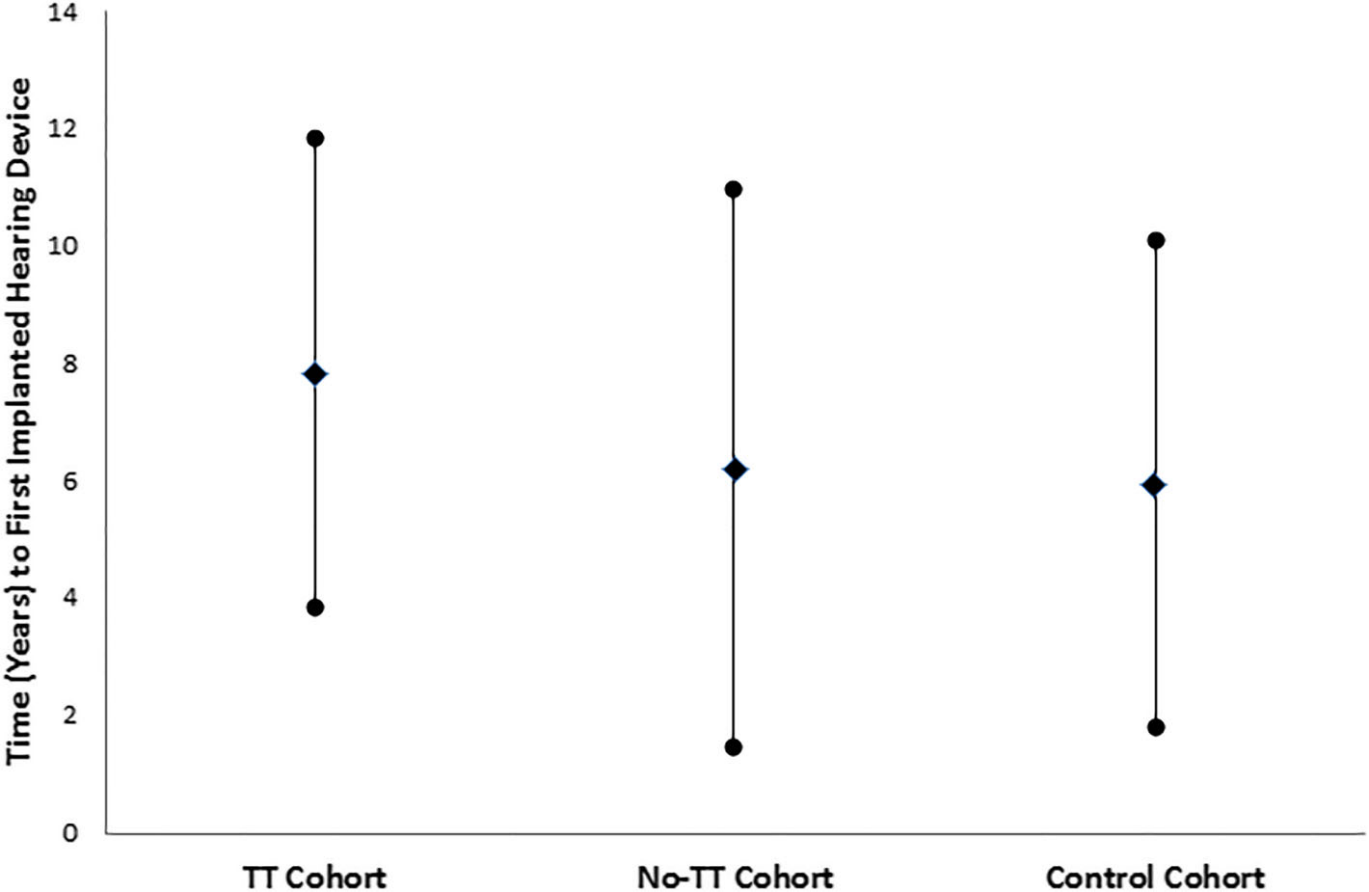
Time (years) (Mean ± SD) from index date to first implanted hearing device (bone-conducting hearing aid) in the three cohorts

Average time from index date to first hearing aid or FM system was found to be above 6 years in all three cohorts (Fig. 1). This analysis was performed only for patients who had an index date of April 1, 1999 or later, as earlier information was not available from the ADP database. Average time from index date to first implanted hearing device (bone-conducting hearing aid) was above 5 years in all three cohorts (Fig. 2).

By cohort, risks of diagnosis of cleft palate/lip were 1.621% (TT cohort), 0.221% (No-TT cohort), and 0.072% (Control cohort). Evaluating the risks of cleft palate/lip in the subgroup of each cohort who obtained “Any Hearing Rehabilitative Device” revealed that only a minority of patients in each cohort who had a hearing device were patients with cleft palate/lip: 6.780% (TT cohort), 2.088% (No-TT cohort), and 1.292% (Control cohort).

## Discussion

In a comprehensive dataset, this study reports the need for assistive hearing devices in ear tube patients, recurrent ear disease patients managed non-operatively, and healthy control patients. Furthermore, there is an association between TT and needing an assistive hearing device. This study is not able to comment on causation, and the authors believe causation should not be inferred.

Controversy exists regarding the role of TT in pediatric patients with recurrent/persistent middle ear disease. While studies support short term resolution of fluid and improvement of hearing in children with otitis media with effusion [9–11], and possibly reduction in episodes of AOM in children with recurrent acute otitis media [12–16], there is also a possibility that the tubes themselves contribute to the development of chronic ear disease and abnormalities of the eardrum [3, 17–19]. Prompt insertion of TT does not improve developmental outcomes in children with persistent otitis media with effusion [20, 21]. Furthermore, children with recurrent middle ear disease who undergo TT placement have higher future risk of advanced ear surgery, compared with those managed conservatively [22]. It is within the context of this controversy that the results of this study, which demonstrated an association between higher assistive hearing device usage in children who have undergone TT placement, are important to consider.

This study demonstrated higher odds ratios of rehabilitative hearing devices in the TT cohort, compared both to the No-TT and Control cohorts. The TT comparison to the Control provides information as to the risk on hearing of the combined effect of the underlying middle ear disease and a possible role of the tubes themselves. The results obtained in this respect are expected, as recurrent/persistent middle ear disease itself is known to be a risk factor for hearing loss [5]. Interestingly, when compared to a group matched for middle ear disease severity (No-TT cohort), there was still a notable increased risk of the need for rehabilitative hearing devices in the children who underwent ear tubes (TT cohort). This data reveals the association of ear tube placement with hearing loss in children with recurrent/persistent middle ear disease. The authors caution that this does not prove causation, but the pattern obtained requires attention. These findings also agree with those obtained by Stenstrom et al. [5] Those authors randomized 113 children with recurrent otitis media with effusion to medical therapy or ear tube placement. They found those children who underwent tubes had hearing thresholds 2.1 to 8.1 dB higher, and much more frequent pathologic abnormalities of the eardum, compared to those treated medically. Similar audiometric and pathologic results were found in other small studies [4, 6, 13, 23]. A large meta-analysis [24] demonstrated that ear tubes increased the long-term risk of eardrum scarring, focal atrophy, retraction, cyst formation (cholesteatoma), and perforation, compared to children not undergoing tubes. Furthermore, the risks were much higher if long-term tubes were used compared with short-term tubes. This last point is particularly interesting, as it further highlights the potential role the tube itself could play.

This study used an operational definition of disease severity. This was the number of presentations to a physician in one year in which the child received a diagnosis of middle ear disease. Both the TT and No-TT cohorts had greater than 5 visits in the year prior to the index date. There is no agreed upon measure of severity of Eustachian tube dysfunction or middle ear disease. The authors believe that within the limitations of administrative data, this measure is the most appropriate and objective measure to match the groups.

A possible explanation for the average 6 year delay (after the index date) in obtaining amplification is that children with mild/moderate hearing loss may not be identified until significant speech and language delays are recognized in school. In Ontario, it is common for children with permanent hearing impairment to be identified to their school boards upon entry to school (age 4–5). Permanent hearing loss may not be identified until academic performance issues arise or behavioral issues are noted. More thorough audiological testing is requested when these issues are identified, and it is at this time that the diagnosis of permanent hearing loss is made, and personal devices (hearing aids and FM systems) are obtained. This delayed identification of permanent hearing loss could account for the 6 year gap in the studied groups. It is furthermore important to note that the differences between the groups in time to first hearing aid/FM system or time to first implanted hearing device are not statistically significant. This prevents further conclusions from being drawn regarding this data.

The strengths of this study include a large comprehensive population-based design, which includes all patients and all surgeons that perform TT in Ontario. Additionally, the ability to use two control groups and have long-term follow-up greatly enhanced the design. The size of the odds ratios between the groups are notable.

Administrative data has limitations. This study design does not permit an assessment of causation, just an association. Factors that could influence the outcomes such as the indications for surgery, indications for choosing conservative management with serial examinations, secondary smoke exposure, daycare attendance, use of pacifiers, developmental delay, family history of otitis media, audiometric testing results, OHIP diagnostic code data quality, and findings at surgery are not available. Data quality is dependent on accurate coding by the physician and hospital coders. A comparison of administrative data with hospital chart data concluded that major events (surgical procedures, mortality, patient demographics, primary diagnoses) are accurately coded [25]. The diagnostic codes used in constructing the No-TT and Control cohorts were the identical codes that were used by physicians coding middle ear disease in the TT cohort children. Although syndromic children and those with craniofacial abnormalities may be more likely to receive TT than to be managed conservatively, the authors believe that minimal bias would be introduced here, as these patients represent a small minority of those who undergo TT. In a cohort of over 200,000 children who underwent TT, Djurhuus et al. [26] found only 0.6% had a diagnosis of cleft palate, the most common craniofacial anomaly associated with Eustachian tube dysfunction. Furthermore, our study demonstrated that amongst the patients in each group who obtained a hearing rehabilitative device, only a minority of patients had a diagnosis of cleft palate/lip. Furthermore, in each cohort, over 95% of the patients with cleft palate/lip did not proceed to a rehabilitative hearing device. As such, we believe the significant difference in rates of rehabilitative hearing devices between the cohorts cannot be adequately explained by a difference in rates of craniofacial abnormalities. Finally, the numbers obtained for FM systems may underestimate the actual incidence, as some schools will purchase FM systems themselves, which would not be captured in ADP data. However, the authors would expect a comparable under-representation of the number of children using FM systems in all three cohorts.

## Conclusions

This study revealed the actual prevalence of hearing assistive device usage in children in Ontario (healthy, middle ear disease with or without tube placement). While the overall risks are low, recurrent/persistent ear disease and ear tube placement are associated with higher risks. This association requires further evaluation in prospectively-designed studies.

## Data Availability

The datasets generated and/or analyzed during the current study are available in the ICES Queen’s Data repository, stored at ICES Queen’s.

## Abbreviations

ADP: Assistive Devices Program
AOM: Acute Otitis Media
FM: Frequency Modulation
ICES: Institute for Clinical Evaluative Sciences
IKN: ICES Key Number
OHIP: Ontario Health Insurance Plan
OME: Otitis Media with Effusion
OR: Odds Ratio
RPDB: Registered Persons Database
TT: Tympanostomy Tube(s)
